# The epidemiology of sickle cell disease in children recruited in infancy in Kilifi, Kenya: a prospective cohort study

**DOI:** 10.1016/S2214-109X(19)30328-6

**Published:** 2019-08-23

**Authors:** Sophie Uyoga, Alex W Macharia, George Mochamah, Carolyne M Ndila, Gideon Nyutu, Johnstone Makale, Metrine Tendwa, Emily Nyatichi, John Ojal, Mark Otiende, Mohammed Shebe, Kennedy O Awuondo, Neema Mturi, Norbert Peshu, Benjamin Tsofa, Kathryn Maitland, J Anthony G Scott, Thomas N Williams

**Affiliations:** a KEMRI-Wellcome Trust Research Programme, Kilifi, Kenya; b Faculty of Medicine, Imperial College, St Mary's Hospital, London, UK; c London School of Hygiene & Tropical Medicine, London, UK; d INDEPTH Network, Accra, Ghana

## Abstract

**Background:**

Sickle cell disease is the most common severe monogenic disorder in humans. In Africa, 50–90% of children born with sickle cell disease die before they reach their fifth birthday. In this study, we aimed to describe the comparative incidence of specific clinical outcomes among children aged between birth and 5 years with and without sickle cell disease, who were resident within the Kilifi area of Kenya.

**Methods:**

This prospective cohort study was done on members of the Kilifi Genetic Birth Cohort Study (KGBCS) on the Indian Ocean coast of Kenya. Recruitment to the study was facilitated through the Kilifi Health and Demographic Surveillance System (KHDSS), which covers a resident population of 260 000 people, and was undertaken between Jan 1, 2006, and April 30, 2011. All children who were born within the KHDSS area and who were aged 3–12 months during the recruitment period were eligible for inclusion. Participants were tested for sickle cell disease and followed up for survival status and disease-specific admission to Kilifi County Hospital by passive surveillance until their fifth birthday. Children with sickle cell disease were offered confirmatory testing and care at a dedicated outpatient clinic.

**Findings:**

15 737 infants were recruited successfully to the KGBCS, and 128 (0·8%) of these infants had sickle cell disease, of whom 70 (54·7%) enrolled at the outpatient clinic within 12 months of recruitment. Mortality was higher in children with sickle cell disease (58 per 1000 person-years of observation, 95% CI 40–86) than in those without sickle cell disease (2·4 per 1000 person-years of observation, 2·0–2·8; adjusted incidence rate ratio [IRR] 23·1, 95% CI 15·1–35·3). Among children with sickle cell disease, mortality was lower in those who enrolled at the clinic (adjusted IRR 0·26, 95% CI 0·11–0·62) and in those with higher levels of haemoglobin F (HbF; adjusted IRR 0·40, 0·17–0·94). The incidence of admission to hospital was also higher in children with sickle cell disease than in children without sickle cell disease (210 per 1000 person-years of observation, 95% CI 174–253, *vs* 43 per 1000 person-years of observation, 42–45; adjusted IRR 4·80, 95% CI 3·84–6·15). The most common reason for admission to hospital among those with sickle cell disease was severe anaemia (incidence 48 per 1000 person-years of observation, 95% CI 32–71). Admission to hospital was lower in those with a recruitment HbF level above the median (IRR 0·43, 95% CI 0·24–0·78; p=0·005) and those who were homozygous for α-thalassaemia (0·07, 0·01–0·83; p=0·035).

**Interpretation:**

Although morbidity and mortality were high in young children with sickle cell disease in this Kenyan cohort, both were reduced by early diagnosis and supportive care. The emphasis must now move towards early detection and prevention of long-term complications of sickle cell disease.

**Funding:**

Wellcome Trust.

## Introduction

Sickle cell disease is a major, but widely neglected, public health issue in low-income countries.^1^ Estimates suggest that, in many parts of Africa, around 1% of all births are affected by sickle cell disease^2^ and the condition causes 6–15% of all deaths in children younger than 5 years.^3^ Haemoglobin S (HbS) has been selected to high frequencies in many populations in tropical areas because of a survival advantage in carriers (with sickle cell trait caused by inheritance of haemoglobin A [HbA] and HbS [HbAS]) against death due to *Plasmodium falciparum* malaria.^4^ However, positive selection for carriers has been balanced by negative selection for homozygotes with sickle cell disease (HbSS), which in Africa is associated with under-5 mortality of 50–90%.^5^

Globally, around three-quarters of all those affected with sickle cell disease live in sub-Saharan Africa, where an estimated 240 000 babies with HbSS are born every year.^2^ Nevertheless, in contrast to high-income regions, few studies have documented the natural history of sickle cell disease in Africa.^6^ Investigation of this natural history is important, as it might differ from other regions because of high proportions of undernutrition, the heavy burden of malaria and other infectious diseases, and more restricted medical care.^2^, ^7^ In this study, we describe the epidemiology of sickle cell disease in early life in a cohort study on the coast of Kenya.

**Research in context**

**Evidence before this study**

We searched PubMed for articles published before Dec 31, 2018, with no language restrictions, with the search terms “sickle cell disease” AND “cohort” AND “Africa”. Of the 402 papers we identified, only one reported on outcomes for a cohort of children recruited from birth. The number of children with sickle cell disease included in this previous report was low, and no detailed data on disease-specific events were reported. After exclusion of 64 reviews and commentaries, most other studies were based on cohorts of older children or adults, people attending outpatient clinics, patients receiving specific treatments, including hydroxycarbamide, or people who were admitted to hospital. Therefore, most existing studies have been small or based on potentially unrepresentative subgroups of patients with sickle cell disease, providing little information about the natural history of the disease from early life. Although several neonate screening projects have recently been established in Africa, we found no publications that reported in detail on subsequent outcomes.

**Added value of this study**

To our knowledge, this study is the first to provide detailed comparative outcome data in a cohort of children both with and without sickle cell disease who were recruited during infancy in an African country. Therefore, it is the first to provide detailed incidence data on a range of outcomes, including death and disease-specific hospital admission.

**Implications of all the available evidence**

This study builds on earlier studies in highlighting the high morbidity and mortality that affect children born in sub-Saharan Africa with sickle cell disease. The study also builds on previous evidence showing that survival among children with sickle cell disease can be substantially improved by early diagnosis and provision of a small range of simple and affordable interventions. This study provides essential scientific data that will be informative to African health ministries and international agencies regarding the burden of morbidity and mortality that falls on children with sickle cell disease and the likely effects of routine interventions.

## Methods

### Study design and participants

This prospective cohort study was done in members of the Kilifi Genetic Birth Cohort Study (KGBCS) on the Indian Ocean coast of Kenya.^8^ Recruitment to the study was facilitated through the Kilifi Health and Demographic Surveillance System (KHDSS), which covers a resident population of 260 000 people and has previously been described in detail.^9^ Rolling recruitment to the KGBCS was undertaken between Jan 1, 2006, and April 30, 2011. All children who were born within the KHDSS area and who were 3–12 months of age during the recruitment period were eligible for inclusion, except for those who had already been recruited to other biomedical studies. The homes of eligible infants were visited (with a maximum of three attempts) by a team who sought consent from their parents, if at home. Capillary blood samples (200–500 μL) were collected into EDTA (edetic acid) and tested for sickle cell disease at the laboratories of the KEMRI-Wellcome Trust Research Programme (KWTRP) in Kilifi (Kenya). Test results, reported as sickle cell disease or no sickle cell disease, were fed back to parents as previously described in detail.^10^ Parents of those who tested positive were offered basic counselling about sickle cell disease, its method of transmission and clinical consequences, confirmatory testing, and a package of basic outpatient care at Kilifi County Hospital, the only provider of specialist services within the region. This outpatient package included parental education regarding sickle cell disease, its method of inheritance and potential consequences, and the provision of pneumococcal prophylaxis with phenoxymethylpenicillin (250 mg once daily until the age of 5 years), malaria prophylaxis with proguanil (3 mg/kg per day), and folic acid supplementation (5 mg once daily), all offered through 3-monthly clinic appointments. Participants who enrolled at the clinic were also issued with emergency cards for fast-track access to the paediatric wards when sick. All infants received routine vaccinations, including the *Haemophilus influenzae* type b conjugate vaccine, which was introduced in Kenya in November, 2001. The ten-valent pneumococcal conjugate vaccine was introduced in Kenya in January, 2011, and was accompanied by a catch-up campaign in Kilifi county for children younger than 5 years. Therefore, most cohort members had received this vaccine but at various ages after recruitment. Hydroxycarbamide therapy was not available at the time of the study. The study population has been previously described in detail;^9^ however, of particular relevance to the current study, levels of education within the Kilifi area are among the lowest in Kenya, with 71% of 15–30 year olds having completed primary education and only 13% having completed secondary education. Only 35% of the population live in semi-permanent or permanent housing of solid construction, and most people follow a lifestyle of subsistence farming under conditions of poor soils and unreliable rainfall.

Written informed consent for inclusion in the KGBCS was obtained from the parents of all participants and ethics permission for the study was obtained from the Kenya Medical Research Institute/National Ethical Review Committee in Nairobi (Kenya) and the Oxford Tropical Research Ethics Committee (Oxford, UK).

### Participant follow-up

We passively followed up study participants for a range of specific outcomes until their fifth birthday. Participant vital status (alive, dead, or outmigrated) was recorded thrice-yearly through linkage to the population register of the KHDSS^9^ and disease-specific hospital admissions were recorded through linkage to an established surveillance platform on the paediatric wards of Kilifi County Hospital.^9^ Briefly, all children who were residents of the study area covered by the KHDSS were identified and linked in real-time to a computerised database at the time of hospital admission. All admitted children were assessed by trained clinicians who recorded data (including admission and discharge diagnoses) on a standard proforma.^9^ Routine laboratory tests were also done at admission, including full blood counts, malaria blood films, and blood cultures.^11^ For the purposes of this study, we used the WHO definition for severe anaemia— a haemoglobin concentration of less than 50 g/L.^12^ For admissions among those with sickle cell disease, specific complications that were not captured routinely through the ward surveillance system, such as painful crisis and hand–foot syndrome, were classified through retrospective examination of hospital notes. Because of this retrospective assessment and the fact that no specific training was offered in advance to the numerous admitting clinicians who worked at the hospital during the study period, these data should be considered as indicative only. At the time of the study, Kilifi County Hospital was the only public hospital providing care for the population of Kilifi county and was the main admission facility for life-threatening illnesses in children.^9^ Sickle cell disease is widely stigmatised within the region,^13^ and at the time of the study no patient support groups or community organisations were active in this area.

### Laboratory procedures

Blood samples taken at recruitment were stored at 4°C and tested for HbS within 5 days of collection using a Bio-Rad Variant Classic high-performance liquid chromatography analyser (BioRad; Hercules, CA, USA). Sickle cell disease was diagnosed if the major Hb peak was HbS and no HbA was present. Confirmatory PCR-based testing was done in all such participants using genomic DNA extracted as described previously.^8^ All inconsistencies between phenotyping and genotyping results were resolved through DNA sequencing (unpublished data). All participants were further genotyped for the common African 3·7 kb α^+^thalassaemia deletion, as described previously.^14^ No other laboratory tests were routinely done on these recruitment samples. All blood tests collected through the paediatric ward surveillance platform were processed as described previously.^9^ Full blood counts were done on a range of automated counters supplied by Beckman Coulter (High Wycombe, UK); reticulocyte counts were generally not included. All assays were done at the laboratories of the KWTRP in Kilifi (Kenya).

### Statistical analysis

Given 120 children with sickle cell disease and 15 500 children without sickle cell disease, each followed up until the age of 5 years, and an expected incidence of death among children without sickle cell disease of 2·5 per 1000 person-years of observation, this study was powered to detect approximately a ten times increase in mortality among those with sickle cell disease, with α of 0·05.

We compared continuous data with parametric (Student's *t*-tests) or non-parametric (Mann-Whitney) tests as appropriate, and proportions were compared using the χ^2^ test. We calculated the incidence of syndrome-specific admission to Kilifi County Hospital from the number of events and the person-years of observation.^8^ We calculated person-years of observation for hospital admission and death on the basis of follow-up times from the date of birth and the date of recruitment, respectively. We derived incidence rate ratios (IRRs) for death and for both overall and disease-specific admission to Kilifi County Hospital with a generalised Poisson regression model, both with and without adjustment for ethnic group, and α-thalassaemia genotype. Survival time in the analysis was based on the child's age, the date of recruitment was considered as the point of entry to risk of death, and the date of birth was considered as the point of entry to risk of admission. We considered the point of exit from risk as the date of death, outmigration, or the participant's fifth birthday, whichever was earliest. As our analysis was done after the last child recruited was at least 5 years old, all cohort members were censored at one of these points. We visually represented results by use of Kaplan-Meier survival curves and Nelson-Aalen cumulative hazards graphs. Children with sickle cell disease were subcategorised on the basis of their HbF at recruitment (above or below the median value for their age, categorised as 3–5 months, 6–8 months, and 9–11 months), their sickle cell disease genotype (HbSS or HbS/β-thalassaemia), and whether or not they registered for management at the sickle cell disease outpatient clinic at Kilifi County Hospital within 12 months of recruitment to the study.

p<0·05 was considered to indicate a significant difference. All analyses were done with Stata version 15.1.

### Role of the funding source

The funder of the study had no role in study design, data collection, data analysis, data interpretation, or writing of the report. The corresponding author had full access to all the data in the study and had final responsibility for the decision to submit for publication.

## Results

**Figure 1:**
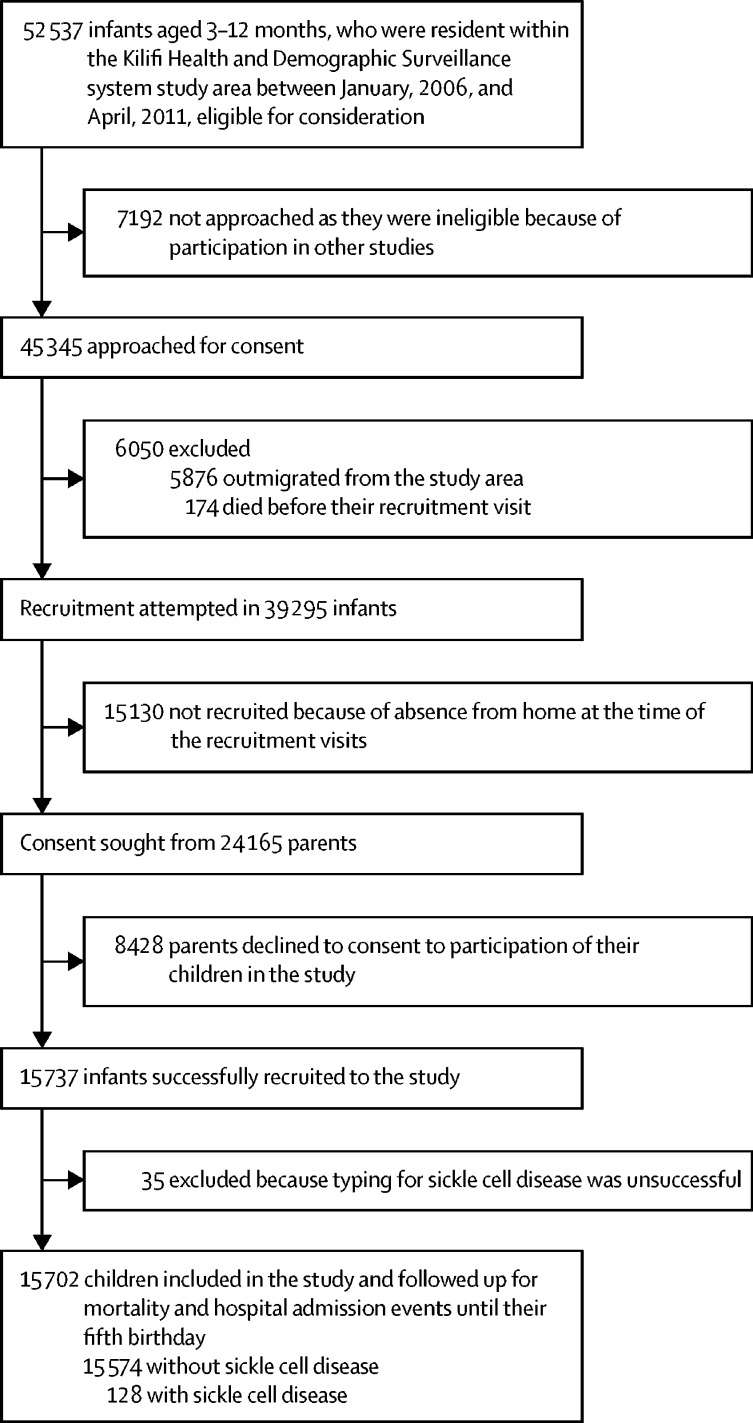
Cohort recruitment and follow-up

In total, 52 537 children were born within the KHDSS area and were aged 3–12 months of age during the recruitment period (Jan 1, 2006, and April 30, 2011; figure 1). 15 737 (30·0%) of 52 537 infants were recruited successfully to the KGBCS at a median age of 6·6 months (IQR 5·1–8·7). The baseline characteristics of recruited and non-recruited children were similar, although a lower proportion of children was recruited from the urban area within the Kilifi township (appendix p 1). 35 (0·2%) of 15 737 successfully recruited patients were excluded from the analysis because insufficient blood was obtained for sickle cell disease testing, leaving 15 702 contributing to the study analysis.

High-performance liquid chromatography analysis was consistent with sickle cell disease in 128 (0·8%) of 15 702 cohort members. Confirmatory testing by PCR showed that 118 (92·2%) of 128 participants were rs334 homozygotes (HbSS) and ten (7·8%) of 128 participants were rs334 heterozygotes. The rs334 heterozygotes were further investigated by sequencing, which showed that all were compound heterozygotes for HbS plus one of three different β^0^ thalassaemia mutations (CD22 [GAA→TAA], initiation codon [ATG→ACG], and IVS1-3′ end del 25 base pairs; unpublished data). Typing for α-thalassaemia was successful in 15 451 (98·4%) of 15 702 participants, of whom 7503 (48·6%) were heterozygotes and 2614 (16·9%) were homozygotes. 127 (99·2%) of 128 children with sickle cell disease were typed successfully for α-thalassaemia, of whom 51 (40·2%) were heterozygotes and 18 (14·2%) were homozygotes. Although the parents of all infants diagnosed with sickle cell disease were informed and invited to enrol their children at the clinic, only 70 (54·7%) of 128 accepted this offer within 12 months of recruitment.

**Figure 2:**
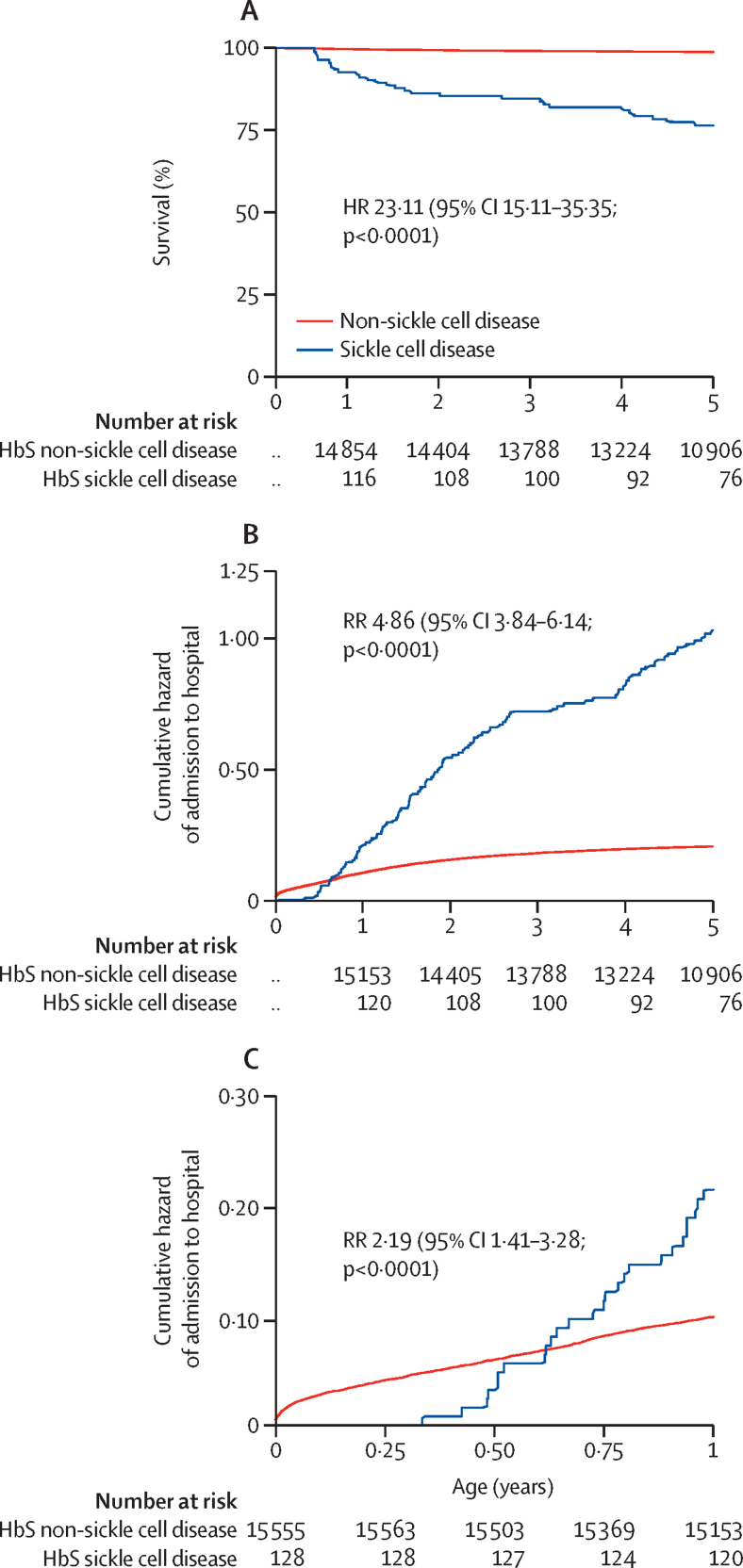
Survival probability and cumulative hazard of admission Kaplan-Meier mortality plot (A) and Nelson-Aalen cumulative hazard plots for admission to hospital over 5 years (B) and 1 year (C) in children with and without sickle cell disease. HR=hazard ratio. HbS=haemoglobin S. RR=rate ratio.

174 participants died before the age of 5 years during a total of 61 446 person-years of observation—a crude mortality rate of 2·8 per 1000 person-years of observation (95% CI 2·4–3·2). There were 26 deaths in 445 person-years of observation among children with sickle cell disease and 148 deaths in 61 001 person-years of observation among children without sickle cell disease, resulting in mortality rates of 58 per 1000 person-years of observation (95% CI 40–86) and 2·4 per 1000 person-years of observation (2·0–2·8), respectively (adjusted IRR 23·1, 95% CI 15·1–35·3; figure 2). Mortality in those with sickle cell disease was significantly lower among those with a recruitment HbF above the age-standardised median versus below the age-standardised median (adjusted IRR 0·40, 0·17–0·94) and among those who enrolled at the sickle cell disease clinic versus those who did not (adjusted IRR 0·26, 0·11–0·62; table 1).

**Table 1:** Deaths among children with sickle cell disease, stratified by various baseline factors

	**Deaths**	**Person-years of observation**	**Incidence per 1000 person-years of observation**	**IRR (95% CI)**	**p value**	Adjusted IRR (95% CI)*	**p value**
**Sickle cell disease clinic enrolment**							
No	18	172	104	1 (ref)	..	1 (ref)	..
Yes	8	273	29	0·28 (0·12–0·65)	0·0031	0·26 (0·11–0·62)	0·0024
**Haemoglobin F**							
Below the median	18	213	85	1 (ref)	..	1 (ref)	..
Above the median	8	232	34	0·40 (0·17–0·92)	0·032	0·40 (0·17–0·94)	0·036
α^+^**thalassaemia**							
Normal	13	186	70	1 (ref)	..	1 (ref)	..
Heterozygous	12	183	66	0·94 (0·43–2·07)	0·89	1·10 (0·46–2·25)	0·80
Homozygous	1	72	14	0·19 (0·02–1·51)	0·11	0·19 (0·02–1·49)	0·11
**β-thalassaemia**							
Without β-thalassaemia	25	408	61	1 (ref)	..	1 (ref)	..
With β-thalassaemia	1	37	27	0·42 (0·05–3·15)	0·40	0·54 (0·06–4·36)	0·56

IRR=incidence rate ratio.

* IRRs adjusted for α-thalassaemia genotype, and ethnic group, except for the α-thalassaemia analysis where adjustment was for ethnic group only.

3146 admissions to hospital were observed in 15 702 cohort members during 70 478 person-years of observation—an overall incidence of 45 per 1000 person-years of observation (95% CI 43–46). Person-years of observation for non-sickle cell disease admissions were 69 959 and for sickle cell disease admissions were 519. 1830 (58·2%) of 3146 admissions were in unique participants, and 1316 (41·8%) occurred as multiple admissions within single individuals (appendix p 2). In participants admitted to hospital, those with sickle cell disease had lower haemoglobin and platelet counts and higher white blood cell counts than did those without sickle cell disease (table 2). Only one positive blood film for *Plasmodium falciparum* was recorded among admitted participants with sickle cell disease, in which the parasite density was significantly lower than that seen in participants without sickle cell disease during the same observation period (table 2). The relative risk of admission to hospital among children with sickle cell disease and children without sickle cell disease varied with age (figure 2). The adjusted IRR was 0·18 (95% CI 0·02–1·2) among children younger than 3 months but increased to 4·7 (3·0–7·4) in children aged 6–12 months. The incidence of admission to hospital was also higher in children with sickle cell disease than in children without sickle cell disease (210 per 1000 person-years of observation, 95% CI 174–253 *vs* 43 per 1000 person-years of observation, 42–45; adjusted IRR 4·80, 95% CI 3·84–6·15; table 3). The most common reason for admission to hospital among those with sickle cell disease was severe anaemia (incidence 48 per 1000 person-years of observation, 95% CI 32–71), and a significantly higher proportion of participants with sickle cell disease than without sickle cell disease were severely anaemic at presentation or received a blood transfusion during their hospital stay (p<0·0001 for both; table 3). Children with sickle cell disease accounted for 20 (17%) of 121 transfusions received by cohort members during the period of observation. The incidence of admission with bacteraemia was six times higher among children with sickle cell disease than among children without sickle cell disease, and the most common organisms isolated were *Streptococcus pneumoniae* (six patients), *Salmonella* spp (four patients), *Enterobacter* spp (two patients), *Acinetobacter* spp (two patients), and *Staphylococcus aureus* (one patient). The incidence rates (per 1000 person-years of observation) for admission with specific syndromes associated with sickle cell disease were painful crises (58 episodes, 95% CI 40–83), hand-foot syndrome (12 episodes, 5–26), strokes (four episodes, 1–15), and osteomyelitis (two episodes, 0–14).

**Table 2:** Clinical and laboratory characteristics of hospital-admitted patients, stratified by sickle cell disease status

	**Non-sickle cell disease (n=15 574)**	**Sickle cell disease (n=128)**	**p value**
All admissions*	3037 (96·5%)	109 (3·5%)	..
Age (months)	11·0 (3·2–22·6)	21·1 (12·5–32·7)	0·22
Weight for age Z score	−1·6 (1·5)	−1·8 (1·0)	0·27
Height for age Z score	−1·3 (1·5)	−1·1 (1·0)	0·13
Haemoglobin (g/L)	10·3 (2·9)	6·4 (2·2)	<0·0001
Mean cell volume (fL)	73·7 (14·9)	78·0 (12·0)	0·0036
White blood cells (× 10^9^ per μL)	12·9 (9·5–17·5)	26·5 (19·4–32·6)	<0·0001
Platelets (× 10^6^ per L)	423 (221)	356 (189)	0·0021
*Plasmodium falciparum* parasites (per μL whole blood)†	60 293 (45 908–79 185)	26 196‡	<0·0001

Data are n (%), median (IQR), or mean (SD). p values were estimated using Student's *t*-test or Mann-Whitney test as appropriate.

* 3146 total admissions.

† Data denote values for the subset of children with positive malaria blood films.

‡ Only one participant with sickle cell disease was admitted to hospital with a positive slide during the period of observation.

**Table 3:** Incidence of admission to hospital with clinical conditions by sickle cell disease status

	**Non-sickle cell disease (n=15 574)**		**Sickle cell disease (n=128)**		Adjusted incidence rate ratio (95% CI)*	**p value**
	Events	Incidence	Events	Incidence		
**Clinical syndrome**						
All cause hospital admission	3037	43	109	210	4·8 (3·8–6·1)	<0·0001
Neonatal conditions†‡	496	413	1	101	0·3 (0·03–1·9)	0·18
Malaria§	241	3	1	2	0·5 (0·1–3·8)	0·53
Severe pneumonia¶	1004	14	24	46	3·8 (2·5–5·8)	<0·0001
Very severe pneumonia¶	518	7	12	23	3·4 (1·9–6·1)	<0·0001
Meningitis or encephalitis‖	1062	15	15	28	2·1 (1·2–3·6)	0·0055
Severe malnutrition**	260	4	6	12	3·6 (1·6–8·1)	0·0020
Gastroenteritis††	600	8	7	13	1·8 (0·9–4·0)	0·095
Jaundice‡‡	110	2	17	33	20·4 (11·5–36·2)	<0·0001
Other	577	8	50	96	12·0 (8·5–16·1)	<0·0001
**Laboratory features and outcome**						
Bacteraemia	364	5	15	29	6·0 (3·5–10·4)	<0·0001
Malaria blood film positive	270	4	1	2	0·5 (0·1–3·5)	0·47
Severe anaemia	83	1	25	48	39·1 (24·5–62·5)	<0·0001
Transfused	101	1	20	38	27·0 (16·3–44·8)	<0·0001
Hospital deaths	47	0·7	4	8	11·0 (3·9–30·9)	<0·0001

Some children contributed data to more than one row. Incidence denotes episodes per 1000 person-years of observation. Person-years of observation for non-sickle cell disease admissions were 69 959 and 519 for sickle cell disease admissions.

* Incidence rate ratios are adjusted for the α-thalassaemia genotype and ethnic group.

† Data for the neonatal period reflect incidence per 1000 person-months of observation; person-months of observation were 15 568 for non-sickle cell disease participants and 128 for sickle cell disease participants.

‡ Admission to hospital within the first 28 days of life.

§ A fever in the presence of *Plasmodium falciparum* parasitaemia at any density in children <1 year old or at a density of >2500 parasites per μL in older children.

¶ Defined as described previously.^1^

‖ Clinical detection of neck stiffness, prostration, or coma (Blantyre Coma Score <5), or a bulging fontanel.

** A mid-upper-arm circumference of ≤7·5 cm in children <6 months or of ≤11·5 cm in children ≥6 months.

†† Diarrhoea (three or more loose watery stools per day) with or without vomiting (three or more episodes per day).

‡‡ Clinical recognition of jaundice by the admitting clinician.

Finally, among cohort members with sickle cell disease, the risk of admission to hospital was associated with several factors. Admission to hospital was lower in those with a recruitment HbF level above the median (IRR 0·43, 95% CI 0·24–0·78; p=0·005) and those who were homozygous for α-thalassaemia (0·07, 0·01–0·83; p=0·035). Conversely, admission to hospital was more frequent in those who enrolled at the sickle cell disease clinic than in those who did not (2·02, 1·10–3·70; p=0.022). We observed no differences by sickle cell disease genotype (HbSS *vs* HbS/β-thalassaemia; table 4).

**Table 4:** Incidence of admission to Kilifi County Hospital among children with sickle cell disease, stratified by baseline factors

	**Admissions**	**Person-years of observation**	**Incidence**	**IRR (95% CI)**	**p value**	Adjusted IRR (95% CI)*	**p value**
**Sickle cell disease clinic enrolment**							
No	23	208	110	1 (ref)	..	1 (ref)	..
Yes	86	311	276	2·16 (1·19–3·91)	0·011	2·02 (1·10–3·70)	0·022
**Haemoglobin F**							
Below the median	74	252	293	1 (ref)	..	1 (ref)	..
Above the median	35	267	131	0·51 (0·29–0·89)	0·018	0·43 (0·24–0·78)	0·0048
α^+^**thalassaemia**							
Normal	59	218	269	1 (ref)	..	1 (ref)	..
Heterozygous	44	212	207	0·96 (0·56–1·65)	0·91	1·09 (0·62–1·89)	0·75
Homozygous	6	83	72	0·26 (0·07–0·87)	0·030	0·07 (0·01–0·83)	0·035
**β-thalassaemia**							
With β-thalassaemia	100	476	209	1 (ref)	..	1 (ref)	..
Without β-thalassaemia	9	43	206	0·90 (0·33–2·42)	0·83	1·08 (0·38–3·06)	0·87

IRR=incidence rate ratio.

* IRRs adjusted for α-thalassaemia genotype and ethnic group.

## Discussion

In this study, we have described under-5 mortality and disease-specific hospital admission in a cohort of children living on the coast of Kenya, with an emphasis on sickle cell disease. Sickle cell disease affected 0·8% of cohort members, in whom admission to hospital and mortality were almost five times and more than 20 times higher, respectively, than in those without sickle cell disease. Furthermore, we observed that survival was significantly higher among children with sickle cell disease who registered for specialist outpatient care. Our study provides a rare description of the clinical epidemiology of sickle cell disease in Africa, where most affected people are born.

Most previous studies investigating the clinical course of sickle cell disease in Africa have been based on cohorts of older people,^15^ patients attending outpatient clinics,^16^, ^17^ or those admitted to hospital.^18^, ^19^, ^20^ Therefore, most studies have involved a selected and potentially unrepresentative subgroup who have survived to the point of diagnosis. Although mortality data have been reported from one previous birth cohort study, the number of children with sickle cell disease included was low, and detailed data on disease-specific events were not reported.^21^ Although several neonatal screening projects have been conducted,^22^, ^23^, ^24^, ^25^ none have reported on detailed outcomes. Although the final number of children with sickle cell disease that were followed up is a limitation of our study, our study had sufficient power to make observations in a number of key areas and with the background of these previous studies, to the best of our knowledge, our study provides the most comprehensive description to date of the epidemiology of sickle cell disease in an unselected cohort of children in Africa.

Although the overall mortality rate in participants with sickle cell disease was 58 per 1000 person-years of observation (95% CI 40–86), this rate varied according to several factors. The mortality rate was more than halved among children who enrolled at the outpatient clinic compared with those who did not, in whom mortality was consistent with our earlier estimate of 50–90% among undiagnosed and untreated children across Africa.^5^ Although this difference could reflect parental attitudes to modern health services, it also suggests that early diagnosis accompanied by the provision of parental education and basic clinical care can lead to substantial reductions in early mortality in Africa, just as was found in studies in the USA more than 30 years ago.^26^ The fact that basic clinical care would be readily affordable to governments in many parts of Africa^27^ supports the implementation of such services more widely. Recently, we illustrated the morbidity and survival benefits that can come with the addition of carefully monitored hydroxycarbamide therapy to a basic package of care.^28^ However, given the complex clinical oversight that normally accompanies the use of hydroxycarbamide, scaling up therapy will be a challenge that will either require substantial investments in equipment and personnel or further studies to confirm the safety of more pragmatic approaches to monitoring.

In agreement with observations from studies in high-income countries,^29^ survival was positively associated with HbF levels. Mortality was almost three times lower in participants with sickle cell disease whose baseline HbF was above the median for their age at recruitment. Furthermore, consistent with previous studies,^30^ we observed a non-significant trend towards improved survival in those with α-thalassaemia.

Although only 0·8% of the cohort had sickle cell disease, children with the condition accounted for 3·5% of all admissions to hospital during the study period. Overall, the incidence of hospital admission in participants with sickle cell disease was 210 per 1000 person-years of observation. However, this incidence varied with age, being lower than that of children without sickle cell disease in the first 3 months of life but higher than that of children without sickle cell disease at older ages. Counterintuitively, this observation suggests that the youngest children with sickle cell disease might be healthier than those without the disease. Three mechanisms could be plausible to explain this observation, all of which relate to malaria. First, almost all mothers of children with sickle cell disease will be carriers of HbAS and will have been naturally protected against pregnancy-associated malaria. Second, based on previous studies, it appears that HbSS red cells are more resistant to malaria than HbAS red cells and might therefore have provided a malaria-protective advantage during pregnancy.^31^ Finally, the rate of decline of HbF is significantly slower in children with sickle cell disease than in those without the disease,^32^ which could have led to further protection against *P falciparum* malaria.^33^ All three of these mechanisms could potentially have resulted in the birth of stronger, healthier babies with a reduced susceptibility to a range of both infectious and non-infectious diseases.^31^

As the study was done in a resource poor region where children commonly die outside health facilities and post-mortem examinations are not routinely available, we were not able to identify the cause of death in most children. Therefore, we could not investigate the association between specific causes of death and individual exposures, such as compliance with particular interventions. Furthermore, because the study was done in the context of routine clinical care and detailed research follow-up of children diagnosed with sickle cell disease was not part of the study design, we do not have data on compliance with routinely prescribed drugs.

The increased admission to hospital observed among older children is likely to be conservative for several reasons. First, the costs associated with admission (including transport, hospital fees, and missed employment opportunities) are a deterrent to many families.^34^ Second, although Kilifi County Hospital is the main provider of medical care within the study area, some children might have sought care at other facilities both within and beyond the study borders. Finally, we will only have captured the most severe clinical events by passive surveillance. Consequently, the full burden of sickle cell disease within our cohort might be considerably higher.

As previously reported,^35^ after an era of unprecedented decline, malaria transmission was low within the study area during the study period and, as a result, few children were admitted to hospital with malaria. Despite this, our study does not suggest an increased susceptibility to malaria among children with sickle cell disease, an observation consistent with previous reports.^36^ Declining malaria incidence probably also accounts for why admission to hospital with severe anaemia was also substantially lower, both in children with and without sickle cell disease, than observed in our previous study at the same hospital.^20^ However, in the current study, anaemia was still an important cause for admission to hospital in those with sickle cell disease, among whom 23% were severely anaemic. Given the strong association between malaria and bacteraemia,^37^ the decline in malaria might also have contributed to the lower incidence of bacteraemia in children with sickle cell disease compared with earlier studies conducted in the same area.^8^, ^20^ However, the roll-out of newer vaccines and the provision of penicillin prophylaxis might also have contributed. Whatever the cause, it seems likely that the falling rate of bacteraemia is a strong driver of improved sickle cell disease survival.

In contrast to our findings for death, admission to hospital among children enrolled at the sickle cell disease clinic was more than twice that of participants who were not enrolled. This could reflect the effect of education, through which children attending the clinic are encouraged to seek urgent treatment in the event of intercurrent illnesses, and the fact that many admissions were also triggered by findings made at routine outpatient visits. Our study did not include the collection of detailed social or economic data that could provide additional explanations for variation in some of our outcomes, something that should be considered in future investigations.

Both the main limitation and the main strength of our study relate to our passive follow-up of cohort participants. Limitations of this approach are that it only provides a minimum estimate of the true incidence of specific complications and that it is not sufficient to capture intervening events in detail. However, this approach was simultaneously a strength of our study in that active surveillance would necessarily have interfered to a greater extent with the natural history of disease, both in children with and without sickle cell disease, and resulted in a more distorted picture of any true differences.

In conclusion, a growing proportion of children with sickle cell disease on the coast of Kenya are surviving, reaching medical attention, and requiring life-long care. Policy decisions regarding how best to diagnose and manage children with sickle cell disease and to prevent complications of the disease should be major priorities for health research in Africa.
