# Supplementary material for: The epidemiology of sickle cell disease in children recruited in infancy in Kilifi, Kenya: a prospective cohort study
Source: Lancet Glob Health. 2019 Aug 23;7(10):e1458–66. doi: 10.1016/S2214-109X(19)30328-6 (PMC7024980; doi:10.1016/S2214-109X(19)30328-6)
Supplement: Supplementary appendix [file mmc1.pdf]

THE LANCET
Global Health

Supplementary appendix

This appendix formed part of the original submission and has been peer reviewed. We post it as supplied by the authors.

Supplement to: Uyoga S, Macharia AW, Mochamah G, et al. The epidemiology of sickle cell disease in children recruited in infancy in Kilifi, Kenya: a prospective cohort study. *Lancet Glob Health* 2019; published online Aug 23. http://dx.doi.org/10.1016/S2214-109X(19)30328-6.

Table S1. Characteristics of children eligible for recruitment to the cohort, stratified by whether or not they were successfully recruited.

Characteristic	Recruited	Not recruited
Follow-up for outcomes of interest		
Duration of follow up for deaths (PYO)	61,446	84,673
Deaths (n)	174	231
Deaths/1000 PYO (95% CI)	2.8 (2.4-3.2)	2.7 (2.3-3.1)
Duration of follow up for admissions (PYO)	70,478	115,683
Admissions (n)	3,146	5,594
Admissions/1000 PYO (95%CI)	45 (43-46)	48 (47-49)
Gender		
Female	7,816 (49.7%)	18,251 (49.6%)
Male	7,920 (50.3%)	18,560 (50.4%)
Area of residence		
Banda ra Salama	882 (5.6%)	1,139 (3.1%)
Chasimba	1,709 (10.9%)	2,119 (5.8%)
Gede	708 (4.5%)	668 (1.8%)
Jaribuni	346 (2.2%)	679 (1.8%)
Junju	1,518 (9.6%)	4,548 (12.4%)
Kauma	621 (3.9%)	1,273 (3.5%)
Kilifi Township	944 (6.0%)	8,200 (22.3%)
Matsangoni	1,295(8.2%)	1,686 (4.6%)
Mtwapa	611 (3.9%)	1,777 (4.8%)
Ngerenya	1,290 (8.2%)	1,825 (5.0%)
Roka	1,326 (8.4%)	2,115 (5.7%)
Sokoke	571 (3.6%)	1,206 (3.3%)
Takaungu Mavueni	1,074 (6.8%)	4,180 (11.4%)
Tezo	1,471 (9.3%)	3,718 (10.1%)
Ziani	1,371 (8.7%)	1,667 (4.5%)

Table S2. The number of admissions among cohort members.

The number of admissions per person	non-SCD	SCD N (%)
0	13245 (85.04)	72 (56.25)
1	1830 (11.75)	29 (22.66)
2	364 (2.34)	12 (9.38)
3	93 (0.60)	9 (7.03)
4	31 (0.20)	3 (2.34)
5	5 (0.03)	2 (1.56)
6	3 (0.02)	0 (0)
7	2 (0.01)	1 (0.78)
9	1 (0.01)	0 (0)
10	1 (0.01)	0 (0)
Total	15575	128

Figure S1. Kaplan Meier survival curves for mortality among children with SCD, stratified by α-thalassaemia genotype.

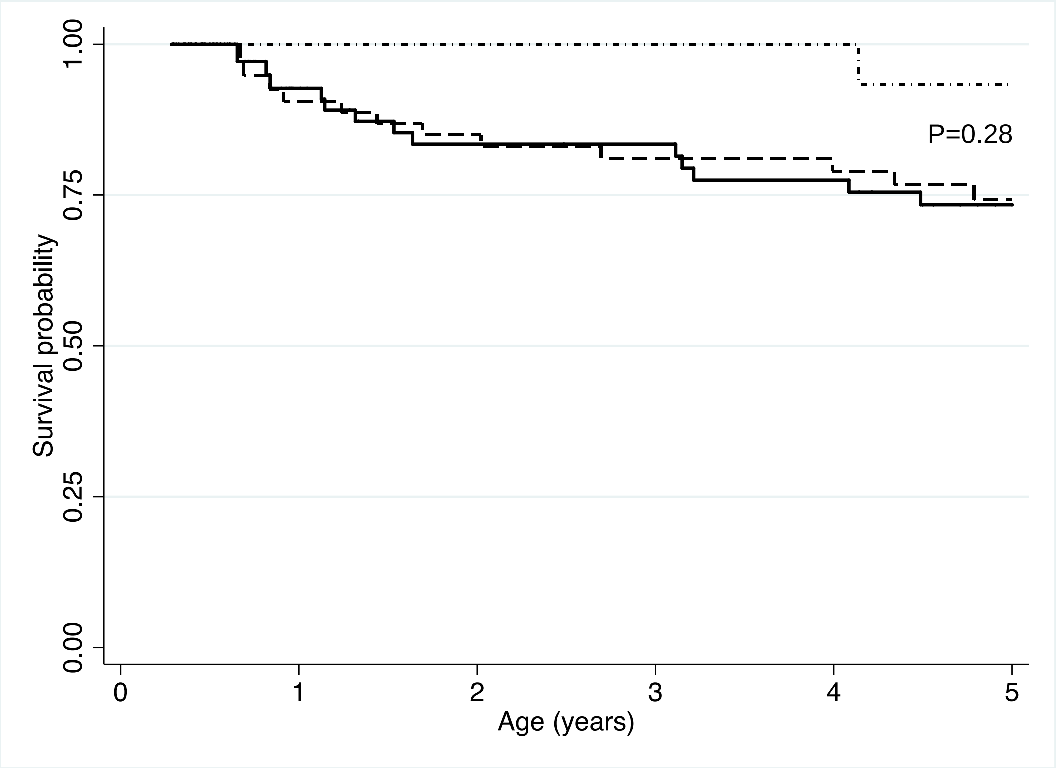

Solid line: no-thalassaemia; dashed line: heterozygous α-thalassaemia; dotted line: homozygous α-thalassaemia. P-value determined by log-rank test for equality of survivor functions. Numbers at risk were: α-thalassaemia normal N=56, Heterozygous N=53 and homozygous N=18.

Figure S2. Kaplan Meier survival curves for mortality among children with SCD, stratified by clinic attendance.

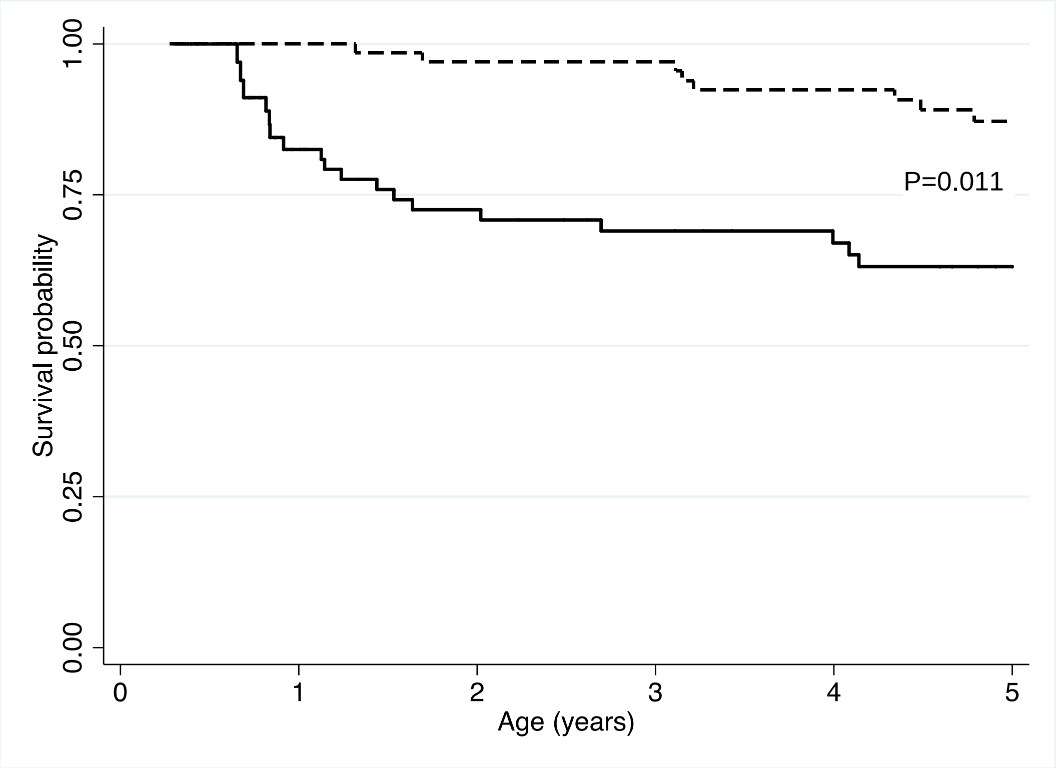

Bold line: not enrolled; dashed line: enrolled. P-value determined by log-rank test for equality of survivor functions. Numbers at risk were: non clinic attenders N=58 and clinic attenders N=70.

Figure S3. Kaplan Meier survival curves for mortality among children with SCD, stratified by HbF levels at recruitment.

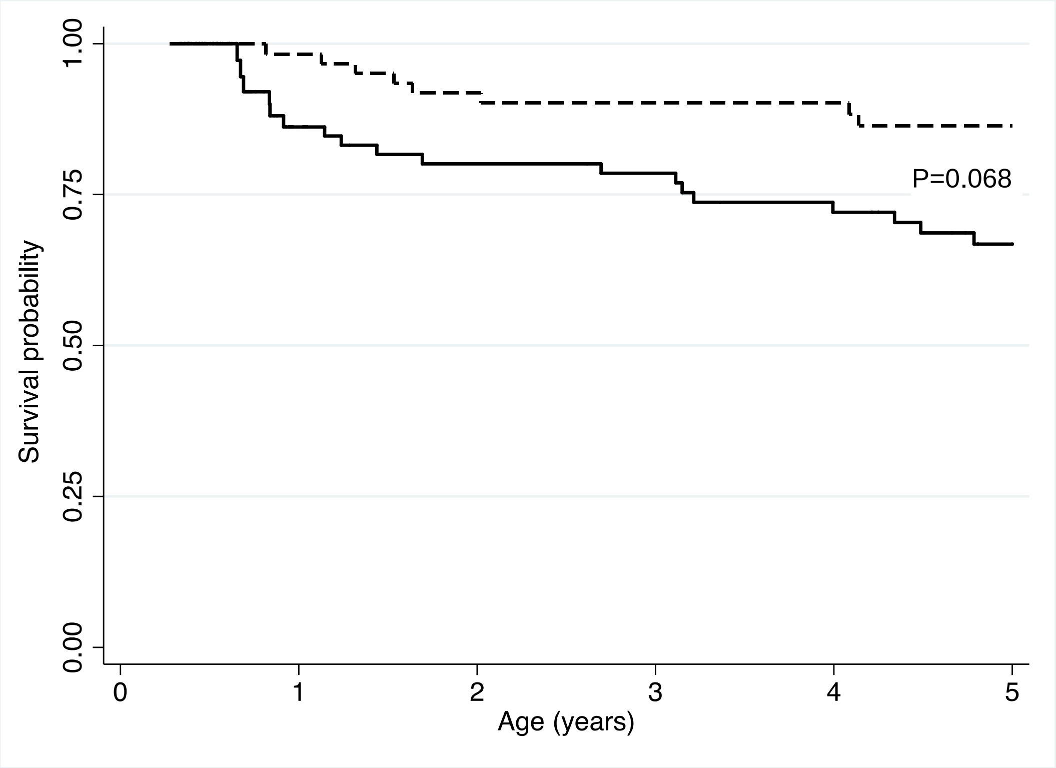

Bold line: below the median; dashed line: above the median. P-value determined by log-rank test for equality of survivor functions. Numbers at risk were: above the median N=64 and below the median N=64.

Figure S4. Kaplan Meier survival curves for mortality among children with SCD, stratified by SCD genotype.

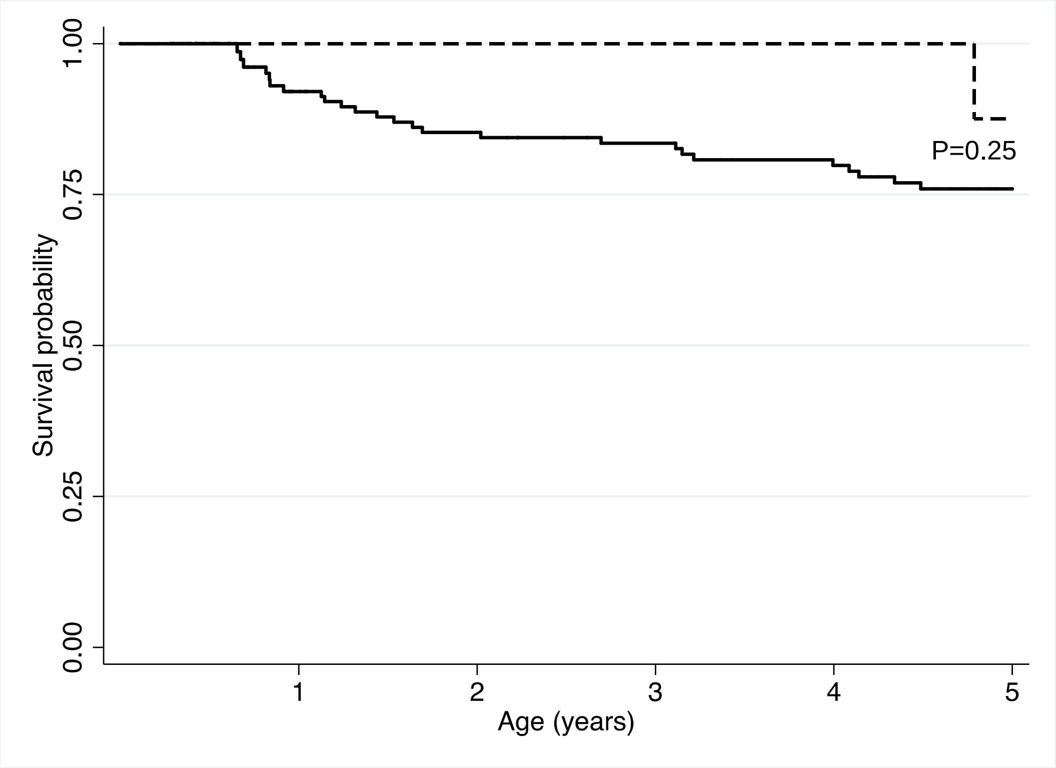

Bold line: HbSS; dashed line: HbS/β^0-thalassaemia. P-value determined by log-rank test for equality of survivor functions. Numbers at risk were: without β-thalassaemia N=118 and with β-thalassaemia N=18.

Figure S5. Nelson-Aalen cumulative hazard estimates for admission to hospital in children with SCD, stratified by α-thalassaemia genotype.

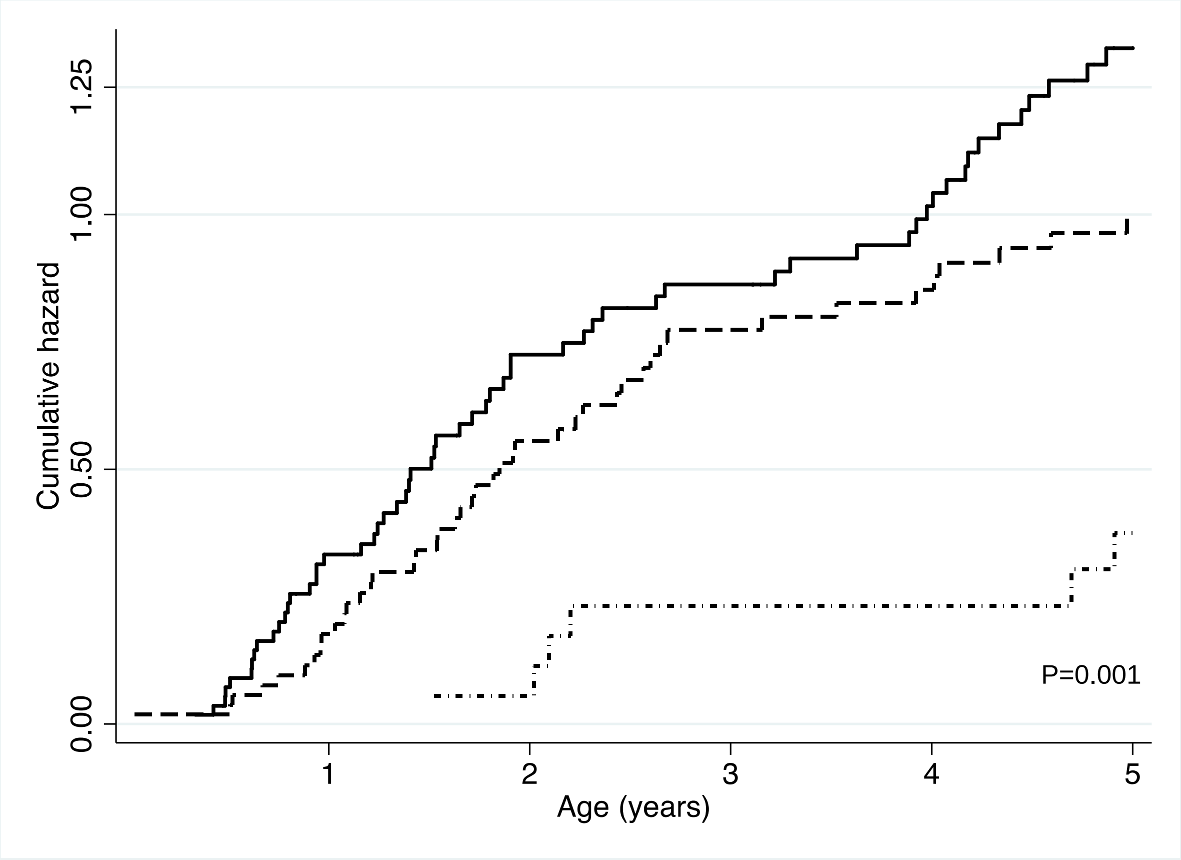

Cumulative hazard for admission by α-thalassaemia genotype: Bold line – no α-thalassaemia; Dash line – Heterozygous α-thalassaemia; Dash-dot homozygous α-thalassaemia. P-value determined by log-rank test for equality of survivor functions. Numbers at risk were: α-thalassaemia normal N=56, Heterozygous N=53 and homozygous N=18.

Figure S6. Nelson-Aalen cumulative hazard estimates for admission to hospital in children with SCD, stratified by enrolment at the SCD clinic.

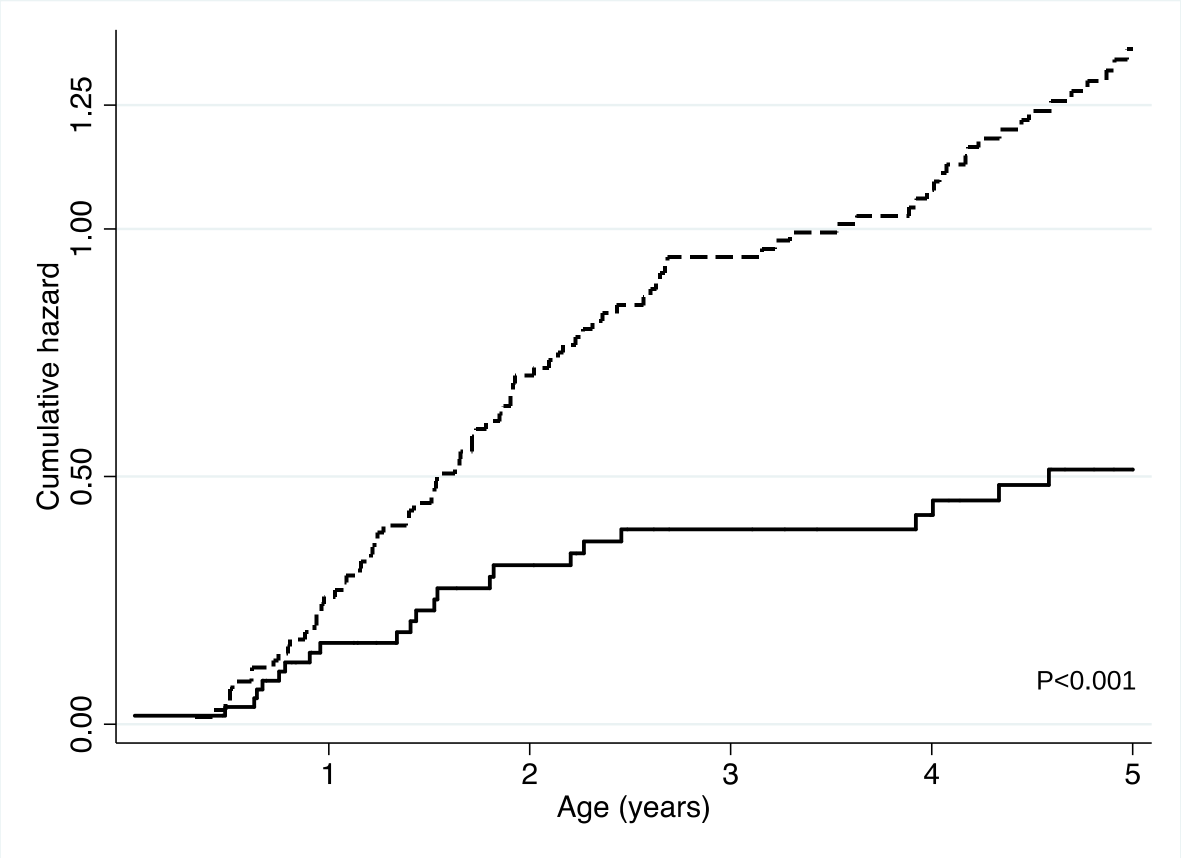

Dashed line – enrolled at the SCD clinic within one year of recruitment; Bold line – not enrolled at the SCD clinic within one year of recruitment. P-value determined by log-rank test for equality of survivor functions. Numbers at risk were: non clinic attenders N=58 and clinic attenders N=70.

Figure S7. Nelson-Aalen cumulative hazard estimates for admission to hospital in children with SCD, stratified by HbF levels at the point of recruitment.

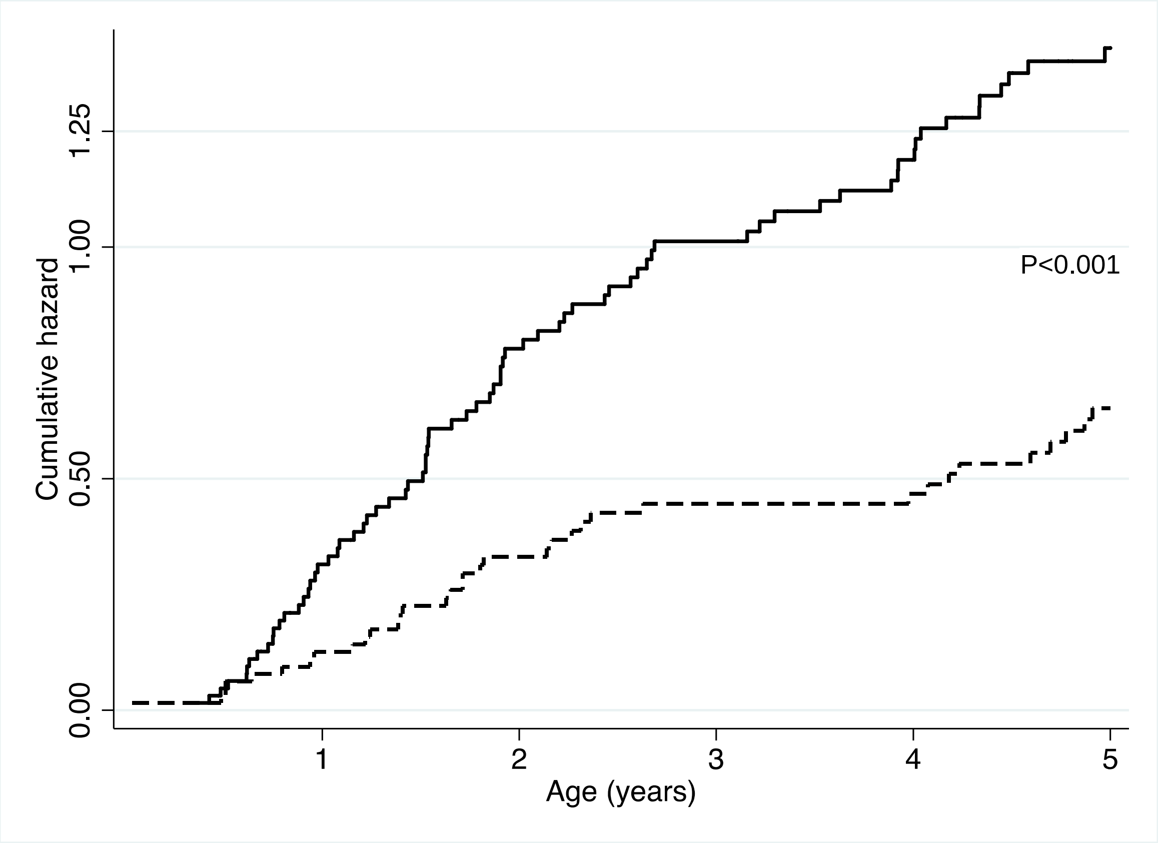

Bold line – below the age-specific median value among children with SCD; Dash line – above the age-specific median value among children with SCD. P-value determined by log-rank test for equality of survivor functions. Numbers at risk were: above the median N=64 and below the median N=64.

Figure S8. Nelson-Aalen cumulative hazard estimates for admission to hospital in children with SCD, stratified by SCD genotype.

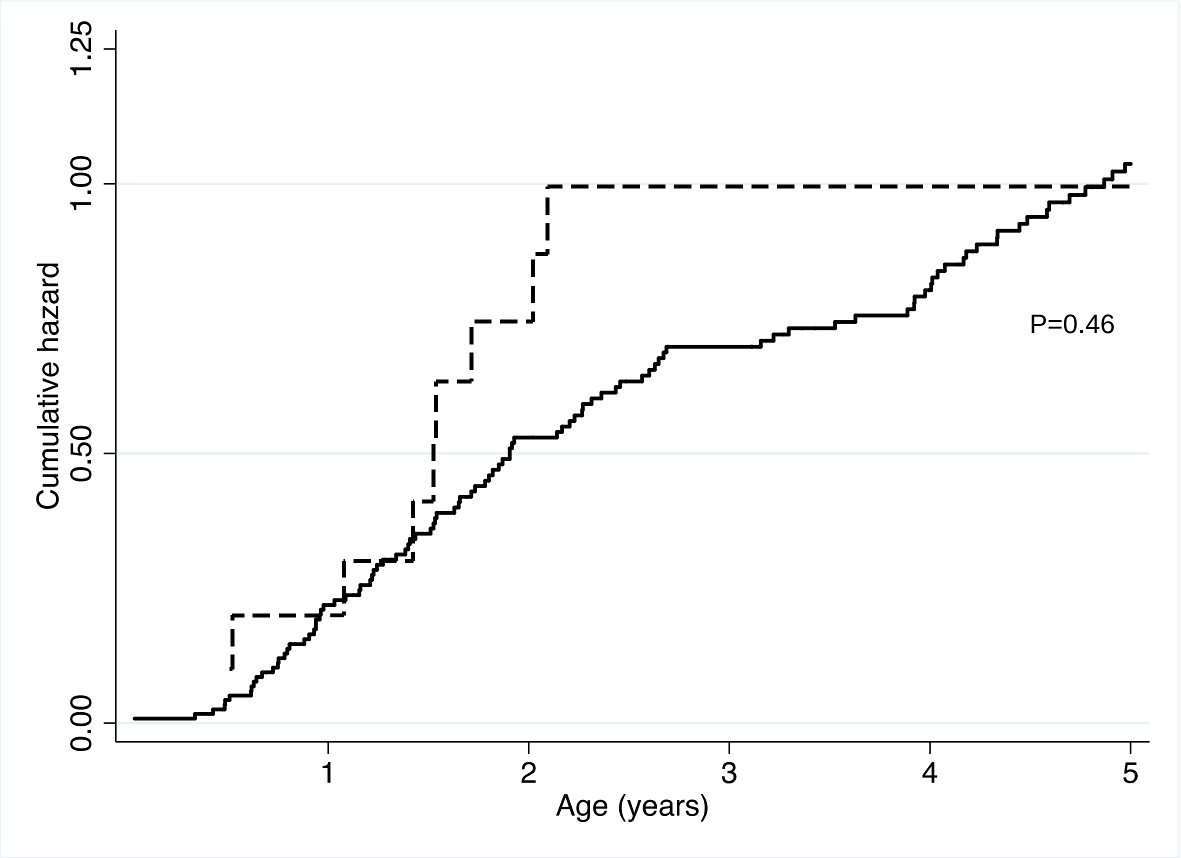

Bold line – SCD HbSS; Dashed line – HbS/β-thalassaemia. P-value determined by log-rank test for equality of survivor functions.
